# Population Genetics Provides Insights Into the Impact of Future Climate Change on the Genetic Structure and Distribution of Asian Warty Newts (Genus *Paramesotriton*)

**DOI:** 10.1002/ece3.71054

**Published:** 2025-04-03

**Authors:** Dung Van Tran, Tomoya Suzuki, Ibuki Fukuyama, Ricardo J. Vera, Kanto Nishikawa

**Affiliations:** ^1^ Graduate School of Human and Environmental Studies Kyoto University Kyoto Japan; ^2^ Wildlife Department Vietnam National University of Forestry Ha Noi Vietnam; ^3^ Graduate School of Global Environmental Studies Kyoto University Kyoto Japan; ^4^ Graduate School of Engineering and Science University of the Ryukyus Okinawa Japan

**Keywords:** Caudata, ecological niche modeling, global warming, *Paramesotriton*, single‐nucleotide polymorphism, Vietnam

## Abstract

Assessing population vulnerability to climate change is essential for informing management and conservation strategies, particularly for amphibians. We integrated population genetics and ecological niche modeling (ENM) to assess the effect of climate change on the distribution and genetic structure of two species of Asian warty newts ( *Paramesotriton deloustali* and *P. guangxiensis* ) in northern Vietnam. We analyzed population genetics using a genome‐wide SNP dataset generated with the MIG‐seq method. Additionally, we applied ensemble ecological niche modeling (ENM) to predict the potential distribution of warty newts under two climate change scenarios (SSP2‐4.5 and SSP5‐8.5) for the periods 2050 and 2090. Population genetics revealed three primary groups: West, East + Cao Bang (CB), and Quang Ninh (QN). CB exhibited discordance between mitochondrial DNA and single‐nucleotide nuclear DNA polymorphism data. Furthermore, gene flow within populations was restricted, particularly within West and QN. Spatial distribution analyses of genetic clusters conditioned by environmental variables predicted that the East + CB genetic cluster would expand, whereas those of West and QN would decrease. The introgression of genetic structures probably reduces the vulnerability of East + CB to climate change. ENM analysis revealed that these newts are susceptible to climate change, resulting in a reduction in their suitable habitat areas across all scenarios. We also observed a shift in the suitable distribution toward higher elevations. Our results suggest that the mountainous areas of northern Vietnam could serve as potential refugia for these newts as the effects of climate change intensify.

## Graphical abstract

Population genetics reveals three groups of Asian warty newts in northern Vietnam. Genetic variation was incorporated in ecological niche models to predict the impact of climate change. Climate change may affect significantly both their genetic structure and distribution.

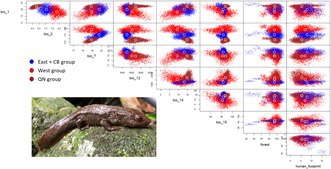

## 1 Introduction

The global climate is undergoing drastic changes, evidenced by a 0.6°C increase in the world's mean temperature over the last century (Jones et al. 2001; Lenssen et al. 2024). Projections also suggest an expected increase of approximately 1.4°C–5.8°C by the end of the 21st century (McKay et al. 2022). Many studies have indicated that climate change is a major threat to global biodiversity (Paterson et al. 2008; Thomas et al. 2004; Trew and Maclean 2021). More than 20% of plant and animal species are projected to face an elevated risk of extinction in response to climate change (Kaeslin et al. 2012). In addition, many species may be forced to adjust their current distributions and adapt to more suitable locations (Chen et al. 2011). Under such conditions, species typically move toward the north (Hannah 2021; Hastings et al. 2020) or relocate to higher elevations (Li et al. 2013; Wilson et al. 2005). Species with limited capacities for dispersal may be more prone to extinction and population decline compared to itinerant species (Duan et al. 2016).

Amphibians may be particularly susceptible to climate change (Foden et al. 2013; Luedtke et al. 2023; Stuart et al. 2004) or land use changes (Hof et al. 2011; Niknaddaf et al. 2023). Luedtke et al. (2023) demonstrated that 39% of amphibian species were impacted by climate change between 2004 and 2022. Amphibians are characterized by limited mobility and a marked sensitivity to variation in environmental temperature and humidity, due to their permeable skin and biphasic life cycle in water and on land (Duellman and Trueb 1994; Stuart et al. 2004). The effects of climate change can alter the geographical distribution and abundance of amphibian populations (Carvalho et al. 2010; Chen et al. 2011), and may also result in a loss of genetic diversity (Abreu‐Jardim et al. 2021). Among amphibians, the urodeles may be particularly vulnerable due to their preference for cooler habitats (Borzée et al. 2019; Sutton et al. 2014; Velo‐Antón et al. 2013; Zhang et al. 2020).

The genus *Paramesotriton* (Asian warty newts; Salamandridae) currently consists of 15 species and is found exclusively in China and northern Vietnam (Frost 2024; Sparreboom 2014). Two species are present in northern Vietnam, *P. deloustali* and *P. guangxiensis* (Sparreboom 2014; Tran, Vu, et al. 2023). Based on a mitochondrial (mt)DNA analysis, Tran, Vu, et al. (2023) identified two genetic groups of *P. deloustali* in the western and eastern parts of northern Vietnam and designated these the West and East groups, respectively. In addition, two separate populations of *P. guangxiensis* have been identified in Cao Bang (CB) and Quang Ninh (QN; Tran, Vu, et al. 2023). However, the genetic structure of Asian warty newts remains unclear. To date, no study has investigated the impact of climate change on the future distribution of species in northern Vietnam. Although several studies have examined the effects of climate change on the distribution of salamanders and newts (Achour and Kalboussi 2020; Ashrafzadeh et al. 2019; Borzée et al. 2019; Ebrahimi et al. 2022; Préau et al. 2020; Zhang et al. 2020), most of these studies have focused on temperate rather than tropical species.

Ecological niche modeling (ENM) is widely used to predict the dynamics of species distributions in response to environmental changes by examining the relationship between current distribution data and environmental variables (Guisan and Thuiller 2005). Such modeling is often performed at the species level (Mota‐Vargas and Rojas‐Soto 2016; Smith et al. 2019). However, species exhibit non‐uniform characteristics across their geographic ranges, with local adaptations occurring along environmental gradients (Hereford 2009; Leimu and Fischer 2008). This intraspecific variation is frequently ignored, potentially leading to over‐ or underestimation in distribution modeling (DeMarche et al. 2019; Mota‐Vargas and Rojas‐Soto 2016; Smith et al. 2019). Integrating genetic structure to include intraspecific variation in ENM can yield more accurate and meaningful predictions, overcoming this limitation (Bayliss et al. 2022; Claerhout et al. 2023; Hu et al. 2021; Zhang et al. 2021). In addition, combining ENM with genetic parameters can enhance our understanding of how environmental variables influence genetic diversity and population genetic structure, as well as provide insights into how populations may respond to rapid climate change in the future (Abreu‐Jardim et al. 2021; Ahmadi et al. 2021; Duan et al. 2016; Milanesi et al. 2018).

The demography and distribution of Asian warty newts in northern Vietnam have been significantly affected by variation in paleoclimate (Tran, Vu, et al. 2023), therefore, we predict that the future climate will also considerably impact both their future distribution and genetic structure. In this study, we evaluated the effects of future climate change on the suitable habitats and genetic structure of Asian warty newts in northern Vietnam. To investigate genetic structure and gene flow, we used genome‐wide nuclear (nu)DNA single‐nucleotide polymorphism (SNP) data of 11 Asian warty newt populations from northern Vietnam. Then, we integrated data on intraspecific genetic variation from different groups into ENM to predict changes in the suitable habitats for each group under two future climate change scenarios: shared socioeconomic pathway (SSP)2‐4.5 and SSP5‐8.5 in two time periods, 2050 and 2090. In addition, we modeled the dynamics of genetic clusters conditioned by climatic variables over time.

## 2 Materials and Methods

### 2.1 DNA Sampling

**FIGURE 1:**
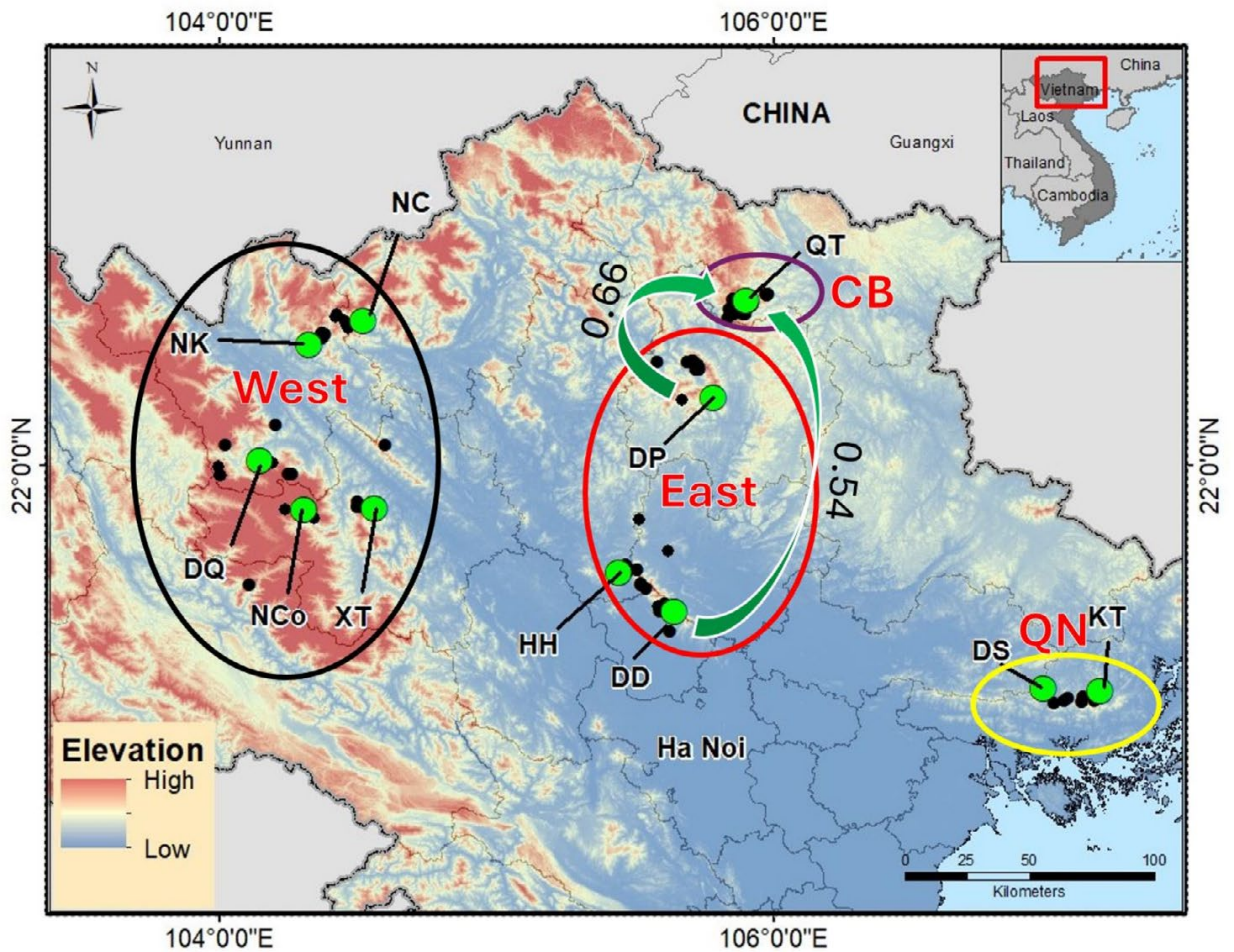
The sampling location for MIG‐seq (green circle) and occurrence location (black circle). The West and East populations were identified as *Paramesotriton deloustali* , while the CB and QN populations were identified as *P. guangxiensis* by the mtDNA analysis. The direction of gene flow among populations (values > 0.1 are shown) estimated by Div‐migrate online is shown by green arrows.

We collected a single piece of tissue (1 × 1 mm) from the tails of each of 62 live newts at 11 locations during field surveys (Figure 1; Table S1). Our field sampling was conducted in 10 selected provinces of northern Vietnam in 2022. Survey transects were established along streams suitable for newts, such as evergreen forests with closed canopies. Each transect was surveyed simultaneously by at least two surveyors (the first author and a field assistant). The species is nocturnal but can also be observed during the daytime (Sparreboom 2014). Therefore, field surveys were conducted during both the day and night. The surveyors walked upstream, carefully searching in a zig‐zag pattern around streams and turning over surface objects (e.g., rocks, leaves, and wood) to locate newts, their larvae, or eggs. Whenever a newt was found, it was caught by hand or with a catching net. The surveyors measured morphological characteristics using calipers, recorded data on geographical coordinates, elevation, time, and microhabitat, and took photographs of living specimens. A tissue sample from the tail of each newt was collected and stored separately in 99% ethanol with a corresponding label. After collecting all necessary data, the newts were released back to their original recorded localities. All animal procedures and specimen collection protocols were carried out in accordance with the animal experimentation guidelines of Kyoto University, Japan (Nos. 20‐A‐7 and 22‐A‐2).

Total DNA was extracted from muscle samples using a DNeasy Blood & Tissue kit (Qiagen, Hilden, Germany), in accordance with the manufacturer's instructions. To evaluate the genetic structure of the populations, we assessed genome‐wide SNPs using multiplexed inter‐simple sequence repeat genotyping and the MIG‐seq method (Suyama and Matsuki 2015). We followed the protocols described by Suyama and Matsuki (2015) and Suyama et al. (2022) for DNA library preparation. DNA libraries from each index sample were pooled and sequenced using the HiSeq platform (Illumina, San Diego, CA, USA). We deposited the raw sequence reads of MIG‐seq data in the DNA Data Bank of Japan (DDBJ) Sequence Read Archive (DRA) under accession number DRA019371 (BioProject ID: PRJDB18910; BioSample ID: SAMD00823858‐SAMD00823919).

### 2.2 Population Genetic Analyses

Quality control for the MIG‐seq data was performed using FASTP version 0.23.2 (Chen et al. 2018) with the default settings. Sequence reads were trimmed to 100 bp, and the short reads were analyzed using a *de novo* assembly pipeline and Stacks software version 2.55 (Catchen et al. 2011). When evaluating SNPs in populations using Stacks, data analysis was restricted to only the first SNP per locus (write‐single‐snp), and loci shared by at least 80% of the individuals across populations were processed (‐R 0.8); sites with less than two minor alleles (min‐mac 2) and > 75% of observed heterozygosity (Ho; max‐obs‐het 0.75) were excluded. We also set the maximum distance allowed between stacks (‐M) to 2, and the minimum depth of coverage to create a stack (‐m) was set to 5 in ustacks. The other data processing parameters were set to default values. We examined the genetic structure of the populations using ADMIXTURE software version 1.3 (Alexander et al. 2009), with the number of clusters (*K*) = 2–11. Cross‐validation error plots were drawn using log data. The results of the admixture analysis were visualized using CLUMPAK (Kopelman et al. 2015; http://clumpak.tau.ac.il). We also performed a principal component analysis (PCA) using TASSEL software version 5 (Bradbury et al. 2007) and visualized the first two eigenvectors in a two‐dimensional plot. Phylogenetic trees for the SNP data were constructed using VCF2PopTree software (Subramanian et al. 2019) and the UPGMA tree algorithm. To estimate the bidirectional migration patterns among populations, we calculated relative migration levels between different sampling sites using the DIV‐MIGRATE online tool (Sundqvist et al. 2016; https://popgen.shinyapps.io/divMigrate‐online/) and GST migration indexes (Hedrick 2005).

The pairwise fixation index (*F*_ST_) (Weir and Cockerham 1984) was calculated from nuDNA SNP data using the function pairwise.WCfst from the hierfstat package (Goudet et al. 2015) in R software version 4.3.0 (R Core Team 2023). As described by Hartl and Clark (1997), *F*_ST_ values were interpreted as follows: values < 0.05 were classified as “little,” 0.05–0.15 as “moderate,” 0.15–0.25 as “great,” and > 0.25 as “very great” genetic differentiation. Correlations between *F*_*ST*_ and geographical distance between populations were analyzed using the Mantel test function in the APE package in R software version 4.3.0, with 9999 permutations (Paradis and Schliep 2019). To quantify the relative contribution of ecological variables to population structure, we modeled the covariance in allele frequencies within and between species and genetic clusters using BEDASSLE v1.6.1 (Bradburd et al. 2013). An additional filter was applied to the SNP dataset, removing individuals with more than 30% missing positions that could affect calculations in VCFtools v0.1.16 (Danecek et al. 2011). Pairwise geographic distances between localities were obtained with the fossil R package (Vavrek 2011) and an ecological distance matrix consisting of elevation, annual temperature, and annual precipitation acquired from WorldClim v2.1 at 30 arcs resolution (Fick and Hijmans 2017) using the terra R package (Hijmans 2025) was included. Models were tested with and without taking overdispersion, or beta‐binomial extension, into account, performing multiple independent short runs of 100,000 generations for parameter tuning, aiming for acceptance rates between 20% and 70%. Final runs consisted of 5 million generations and 20% burn‐in, and model adequacy was evaluated with posterior predictive sampling, calculating the *p*‐value based on the empirical cumulative probability of observed data as described by Bradburd et al. (2013). We also calculated the genetic diversity of each newt population, including *Ho*, expected heterozygosity (*He*), nucleotide diversity (*Π*), and inbreeding coefficients (*F*_IS_) using the populations program in Stacks (Catchen et al. 2011).

### 2.3 Predicting the Dynamics of Genetic Clusters

To predict the dynamics of genetic clusters conditioned by climate change scenarios, we implemented spatially explicit simulations using POPS software (Jay et al. 2015). This software categorizes samples into genetic clusters according to genetic ancestry and incorporates climatic and landscape variables in these cluster assignments. Then it forecasts changes in genetic structure in response to environmental changes (Jay et al. 2012). We simulated genetic clusters under present conditions with the same climatic variables for ENM. To define the number of clusters (*K*), we ran our simulations four times with *K* = 2–11. We selected the *K* values corresponding to the lowest deviance information criterion (Spiegelhalter et al. 2002). The Markov Chain Monte Carlo runtimes in POPS were set to 1200 sweeps, and the burn‐in period was 200 sweeps, in accordance with the software recommendations. Finally, we predicted the spatial changes for each cluster under the following future climate change scenarios in 2050 and 2090: SSP2‐4.5 and SSP5‐8.5.

### 2.4 Predicting the Changes in Suitable Distribution

We determined the occurrence of Asian warty newts in northern Vietnam based on our field surveys (see details in the DNA sampling sections) and published documents. After randomly thinning the presence localities to areas with radii of 1 km to prevent autocorrelation of the occurrence data, we gathered population data from 105 localities (Tran, Vu et al. 2023). Climate variables were acquired from the WorldClim database version 2.1, with a spatial resolution of 30 arcs (Fick and Hijmans 2017). The dataset included 11 layers for temperature and eight layers for precipitation. Elevation data were obtained from the EarthEnv website (https://www.earthenv.org/topography; Amatulli et al. 2018). We also downloaded variables for fractions of forest in each grid cell (Li et al. 2016) and human footprint data (Venter et al. 2016, 2018) to represent land use and human impact, respectively. To address correlation among variables, we computed Pearson's correlation coefficient (*r*) and systematically removed one variable from each highly correlated pair (|*r|* > 0.80). Then, we also utilized the usdm package (Naimi et al. 2014) in R software version 4.3.0 to compute Variance Inflation Factors (VIFs) for our predictors (Table S2). A stepwise process was then applied to select variables with VIF values < 5, as values exceeding this threshold signal a potential risk of strong collinearity (Turner et al. 2019). Finally, we used the following seven variables to run our models: annual mean temperature (Bio1), mean diurnal range (Bio2), temperature annual range (Bio7), annual precipitation (Bio12), precipitation seasonality (Bio15), human footprint, and fraction of forest.

To project future population distributions, we evaluated two SSP scenarios (SSP2‐4.5 and SSP5‐8.5) in two periods: 2050 (the mean of 2041–2060) and 2090 (the mean of 2081–2100) of the global climate model ACCESS‐CM2 (Bi et al. 2020). These models were chosen because they have previously been used to investigate the impact of climate change on organisms in northern Vietnam (Vu et al. 2020; Trinh et al. 2022; Tran, Hoang, et al. 2023). We kept non‐climatic variables unchanged in the climate change projections due to the unavailability of data.

The biomod2 package (Thuiller et al. 2016, 2023) in R software version 4.3.0 (R Core Team 2023) was used to predict the suitable distributions of three groups (West, East + CB, QN). Based on the population genetic analysis of SNP data, we treated East and CB as a single group. Based on preliminary analyses for 10 algorithms in the biomod2 package, the final models were assembled from the following five algorithms: flexible discriminant analysis (FDA), generalized boosted models (GBM), multivariate adaptive regression splines (MARS), maximum entropy (MaxEnt), and random forest (RF). Since presence‐absence data are necessary for most models, with the exception of MaxEnt, we generated pseudo‐absence localities randomly using the BIOMOD_Formatting Data function within the BIOMOD2 package (Thuiller et al. 2016, 2023). The number of pseudo‐absence points created was 10,000 points, aligning with the default setting of background points in the MaxEnt model. Additionally, we conducted 10 repetitions, following the recommendation of the BIOMOD2 team (Thuiller et al. 2023). Subsequently, we partitioned the data into model calibration (80%) and model testing (20%) datasets. We used Cohen's kappa (KAPPA), the area under the receiver operating characteristic curve (AUC), and the true skill statistic (TSS) to assess the performance of each model. The ensemble map of suitable distribution had a logistic output value from 0 (unsuitable) to 1 (highly suitable). In the study, we used the get prediction function in the BIOMOD2 package to generate binary suitability maps of each group. The suitable habitat maps were used to calculate the difference between the future and present suitable areas. The function BIOMOD_RangeSize in the BIOMOD2 package reveal the percent of range loss and range gain, and stable area for each warty newt group.

To assess the trend of shift in suitable distribution, we compared the centroids of suitable habitat distribution in current condition and future scenarios using SDMtoolbox 2.0 (Brown et al. 2017). Additionally, we also assessed the elevational shift and calculated the elevation of each cell within the suitable distribution, then compared the average values between current and future time periods (Wang et al. 2024). The elevation values were extracted from the Terrain Elevation Data 2010 (GMTED2010) by USGS EROS (http://eros.usgs.gov/#). All calculations were implemented in ArcMap 10.7 (ESRI).

### 2.5 Niche Comparison

We applied n‐dimensional hypervolumes (Blonder et al. 2014) to quantify the realized niche space for the different groups (West, East + CB, QN). The Hypervolume package (Blonder and Harris 2018) was used to calculate the shape and volume of hypervolumes for each group using the Gaussian method. The size of the realized niche in multidimensional space was assessed by measuring each hypervolume. We should note that the volume of a hypervolume is quantified as a measure without units. We evaluated niche overlap among the different groups using the BAT package (Cardoso et al. 2021). We measured differentiation between hypervolumes by evaluating both niche shifts, which involved replacing space between hypervolumes, and also niche contraction/expansion, which was the net difference between hypervolumes (Carvalho and Cardoso 2020; Mammola and Cardoso 2020). Niche differentiation ranges from 0 (indicating complete overlap between hypervolumes) to 1 (indicating total separation between hypervolumes; Mammola and Cardoso 2020). We also calculated the Sorensen similarity index among hypervolumes to reveal the similarity of the hypervolumes of the newt groups. The highest Sorensen similarity index is 1 (identical hypervolumes), while the lowest value is 0 (completely dissimilar hypervolumes).

## 3 Results

### 3.1 Population Structure and Migration Between Populations

**FIGURE 2:**
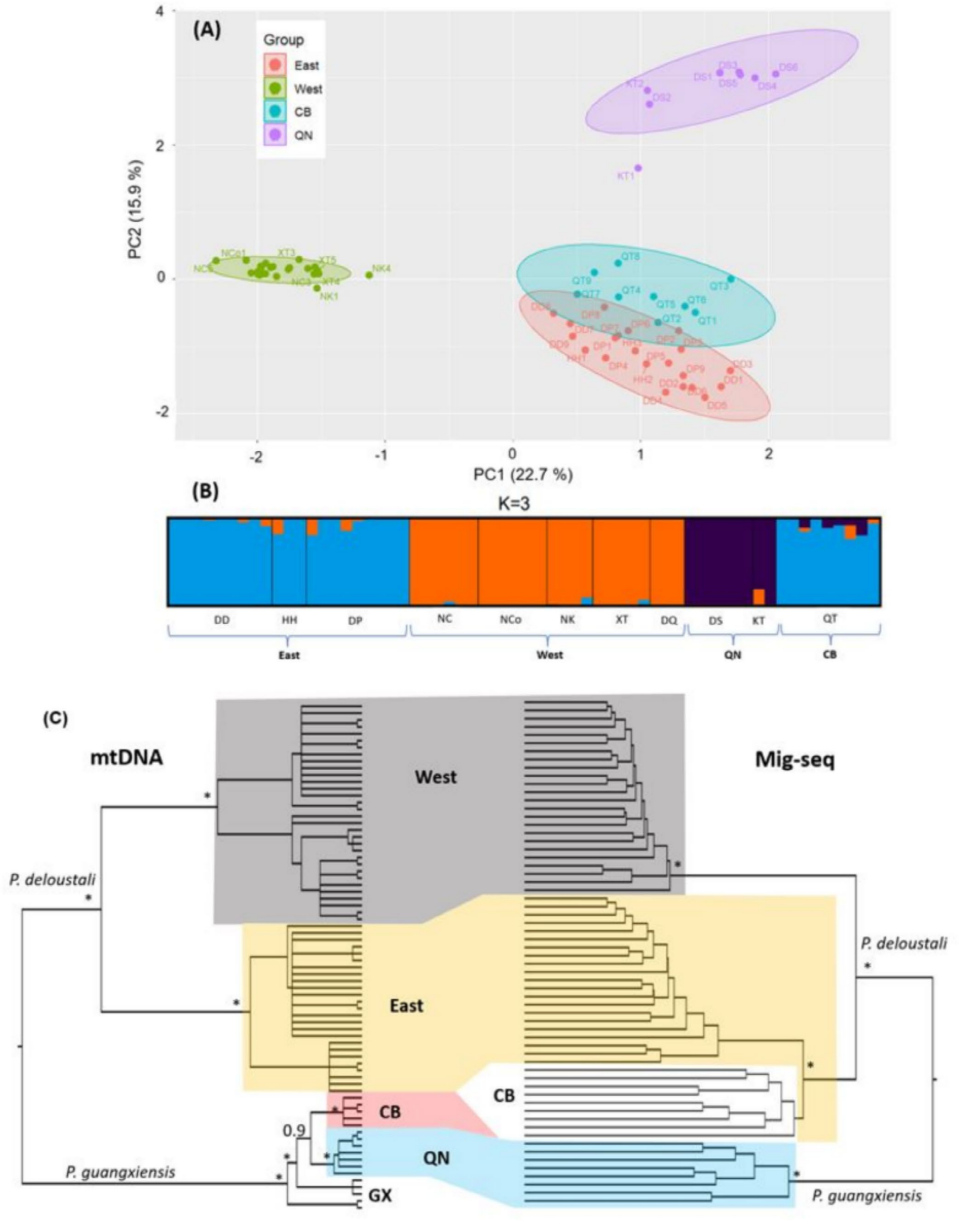
Results of the (A) PCA analysis of PC1 vs. PC2 with 95% confidence ellipses, (B) ADMIXTURE analysis for *K* = 3, and (C) Two phylogenetic trees constructed from mtDNA and nuDNA (MIG‐seq) showed the discordance of the CB population in relationship with other populations of Asian warty newts in northern Vietnam. The mtDNA tree was modified from Tran, Vu, et al. (2023). The nuDNA tree was estimated by VCF2PopTree software (Subramanian et al. 2019). Asterisks indicate adequate statistical support. The GX group was not included in the nuDNA tree.

MIG‐seq analysis generated 381 loci consisting of 52,091 sites after filtering. Mean locus length was 127 bp. After filtering, we retained 205 variant sites. In PCA analyses, the newts clustered into three distinct groups. Samples from DD, DP, and HH primarily clustered together as the East group, whereas those from QT (the CB group) partially overlapped East. The samples from NC, NCo, NK, DQ, and XT clustered as the West group. DS and KT individuals tended to form a separate group (QN; Figure 2A). The mtDNA analysis by Tran, Vu, et al. (2023) identified the samples in DD, DP, HH, NC, NCo, NK, DQ, and XT as *P. deloustali* , whereas the remaining samples (DS, KT, and QT) were *P. guangxiensis* . ADMIXTURE analysis showed the smallest cross‐validation error for the model with *K* = 3 (Table S3; Figure S1). The bar plot mainly corroborated the findings of the PCA. In particular, East + CB, West, and QN were clearly separated. Furthermore, phylogenetic trees constructed from mtDNA and nuDNA SNP data revealed discordance in CB. In the mtDNA tree, CB clustered with QN, identified as *P. guangxiensis* (Figure 2C). Conversely, in the nuDNA tree, CB clustered with East (Figure 2C).

**FIGURE 3:**
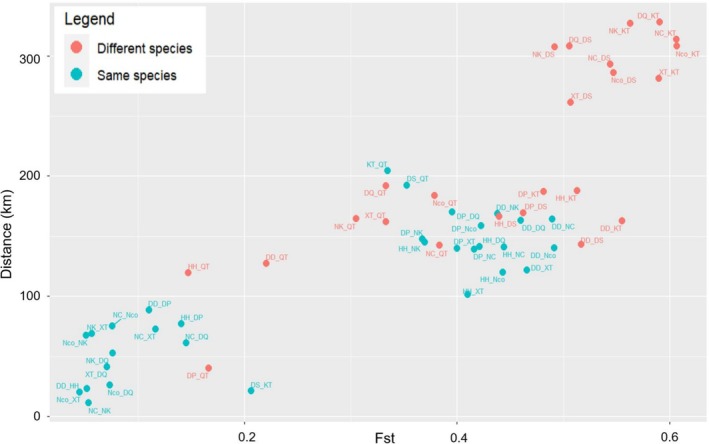
Relationship of the *F*_ST_ and geographic distance between populations of the Asian warty newts in northern Vietnam.

In addition, we estimated *F*_*ST*_ values for populations from different species ( *P. deloustali* and *P. guangxiensis* ) based on nuDNA SNP data. These values ranged from 0.147 to 0.606, whereas those for populations of the same species ranged from 0.045 to 0.491. Notably, QT (part of the CB group) had low *F*_ST_ values compared to the others (East; Figure 3; Table S4). By grouping the populations into main groups (West, East, CB, and QN), we found that the *F*_ST_ values among the groups ranged from 0.19 to 0.54 (Table S5). Among these, the *F*_ST_ value for the East and CB pair was the smallest, while the highest value was observed for the West and QN pair. The Mantel test showed that the populations of *P. deloustali* were significantly isolated by geographic distance (*p* < 0.0001).

BEDASSLE simulations showed poor fit of the observed *F_ST_*, except for the within‐species analysis that accounted for the beta‐binomial extension and obtained low to moderate *p*‐values (Figure S2). However, posterior predictive sampling evidenced that this better fit represented only a subtle improvement (Figure S3). Among all the tests, only the contribution of IBE to the genetic differentiation of *P. delousteli* could be modeled, although with little confidence and recovering small mean aE:aD ratios: (a) elevation, 1.198 × 10^−2^; (2) annual temperature, 3.158; and (3) annual precipitation: 0.125 (Table S6). This would imply in the first case that the effect of 100 m of elevation would be the equivalent of 1.198 km of lateral distance, implying a weak influence of IBE in the population structure of *P. delousteli*.

The divMigrate analyses only found significant gene flow from the DD (0.54) and DP (0.66) populations in East to the QT population in CB (Figure 1). In addition, we observed no significant migration of populations between West, East, and QN (Figure 1).

### 3.2 Genetic Diversity

**FIGURE 4:**
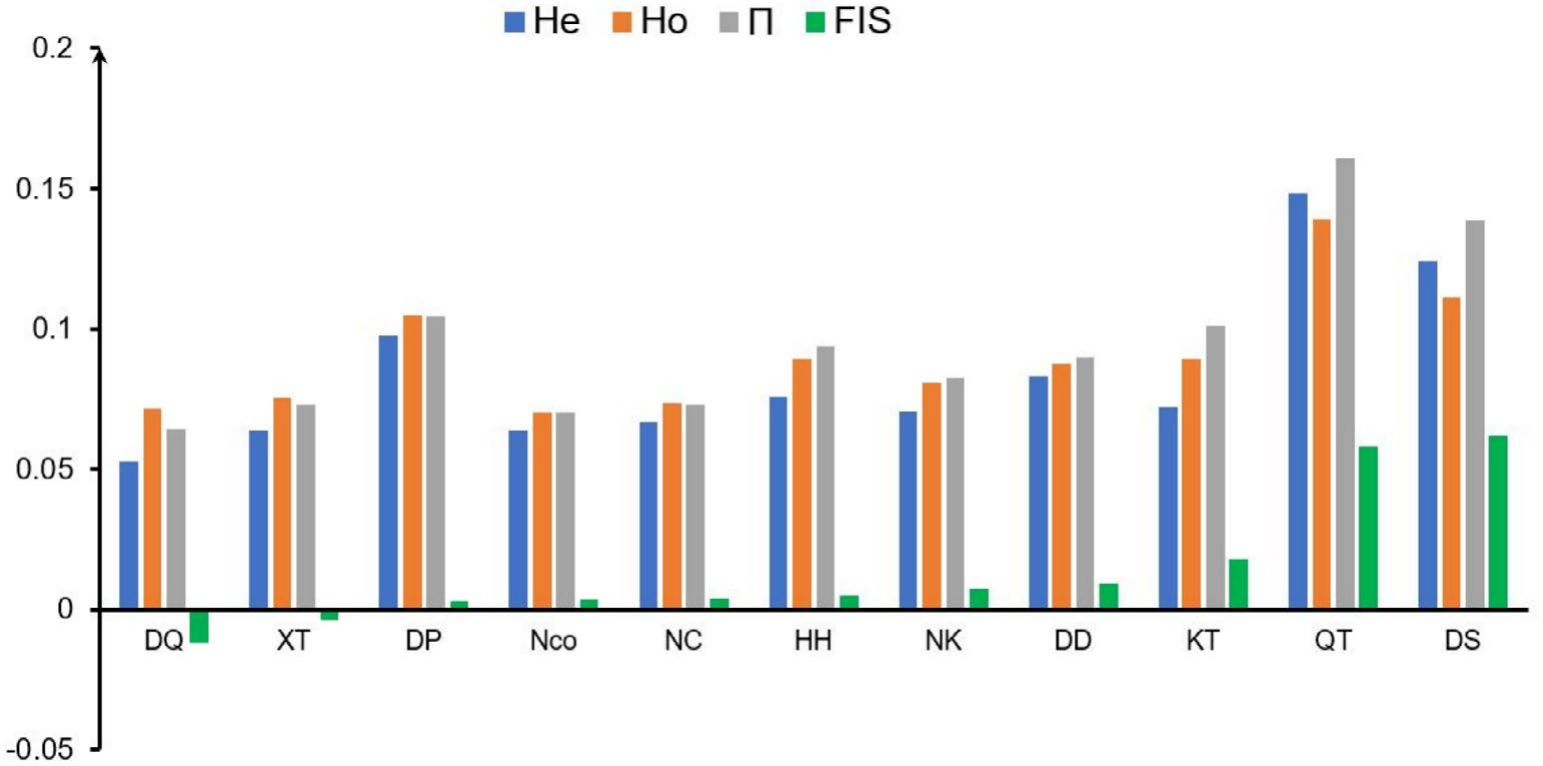
The diversity genetics across loci in each population of Asian warty newts (*He*: Heterozygosity expected under Hardy–Weinberg equilibrium; *Ho*: Observed heterozygosity; *Π*: Nucleotide diversity; *F*_*IS*_: Inbreeding coefficient).

Genetic diversity measures (*He*, *Ho*, and *Π*) were highest for CB, followed by QN, West, and East (Figure 4). The *He* values ranged from 0.05271 (DQ population) to 0.14845 (QT population). The lowest *Ho* value was 0.06367 (NCo population), whereas the highest was 0.13926 (QT population). The highest *Π* value was 0.16077 (QT population), whereas the lowest was 0.06439 (DQ population).

In addition, we found that the *Ho* values were consistently higher than the *He* values across all populations except DS (QN group) and QT (CB group). The F_IS_ values were lower than 0.15 for all populations. An *F*_*IS*_ value above 0.15 suggests considerable inbreeding within a population (Hartl 1988; Wright 1965). *F*_*IS*_ estimates were negative for the DQ and XT populations, with the highest values in the DS (0.06189) and CB (0.05816) populations, and the lowest value in the DQ population (−0.01165; Figure 4).

### 3.3 Predicting Dynamics in Genetic Clusters

**FIGURE 5:**
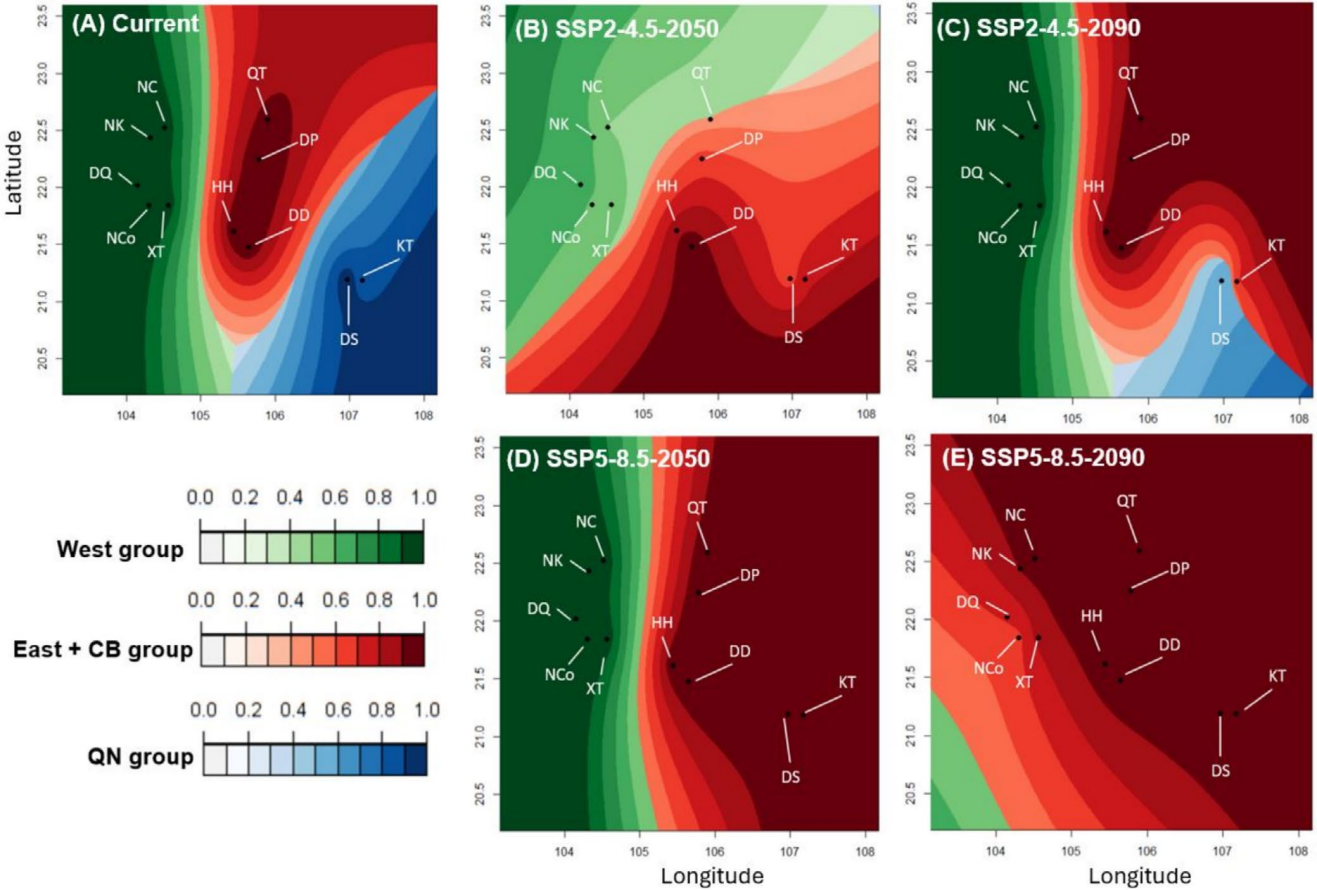
The pattern of genetic clusters conditioned to climatic variables projected for West, East + CB, and QN of Asian warty newts under scenarios of climate change SSP2‐4.5 and SSP5‐8.5 in 2050 and 2090 (Each color set represents a cluster in the figure legend; the black circle shows locations of examined populations).

Our analysis revealed three primary clusters among 11 populations of Asian warty newts, correlated with climatic variables (*K* = 3, deviance information criterion = 7252.85; Figure 5). The correlation between estimated and predicted admixture coefficients for environmental variables was 0.979, indicating high accuracy. In the current climate, the POPS analysis identified Cluster 1, including populations in DD, HH, DP, and QT. Cluster 2 included populations in NC, NK, NCo, DQ, and XT, whereas Cluster 3 included populations in DS and KT. This pattern closely aligned with the results of the admixture analysis: Cluster 1 = East + CB; Cluster 2 = West; and Cluster 3 = QN. Overall, the distribution of the three clusters was predicted to be susceptible to climate change. East + CB exhibited a trend toward expansion across all scenarios, whereas QN faced a decline in three of the four scenarios. The West group was predicted to remain stable in scenarios SSP2‐4.5‐2090 and SSP5‐8.5‐2050 but to decline in the two remaining scenarios.

### 3.4 Niche Differentiation

**FIGURE 6:**
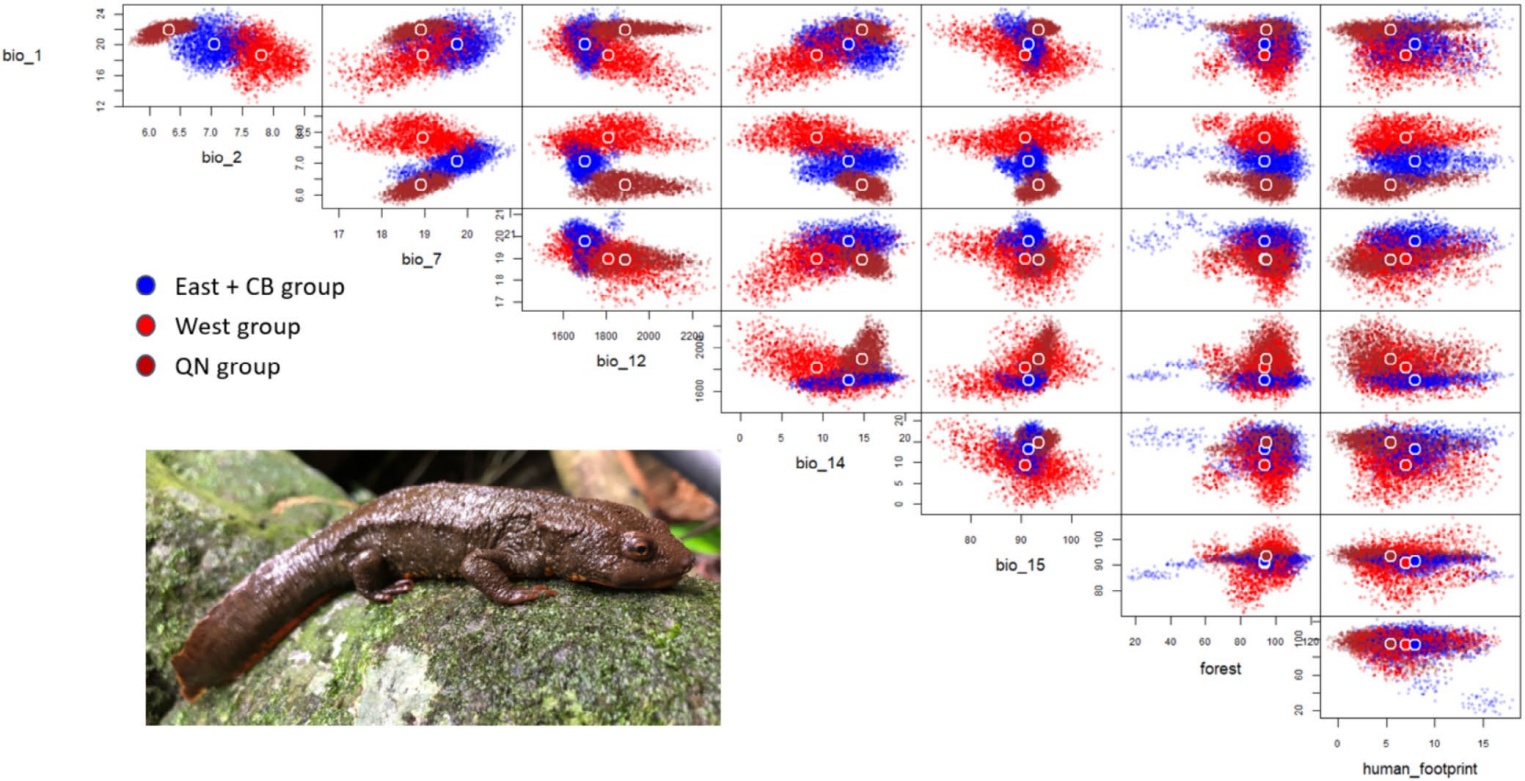
The 10‐dimensional hypervolume for West, East + CB, and QN groups of Asian warty newts in northern Vietnam. A set of 10,000 randomly sampled points was employed for each hypervolume to delineate their boundaries and shapes.

The predicted volume of the realized niche was largest for West (231,604,868), followed by East + CB (11,929,912), and QN (1,867,106) (Figure 6). Niche differentiation was entirely distinct among all comparison pairs. This dissimilarity was primarily due to niche breadth (> 70%), with the contribution from niche shifts being limited (< 30%; Table S3). The intersection index among groups showed the largest overlap between West and East + CB, whereas the smallest overlap was between East + CB and QN. The Sorensen similarity index also present very small similarity among the hypervolume of the newt groups (< 0.1; Table S7). These results indicate that the suitable distribution of the groups should be modeled separately.

### 3.5 Effects of Climate Change on Suitable Distribution

**FIGURE 7:**
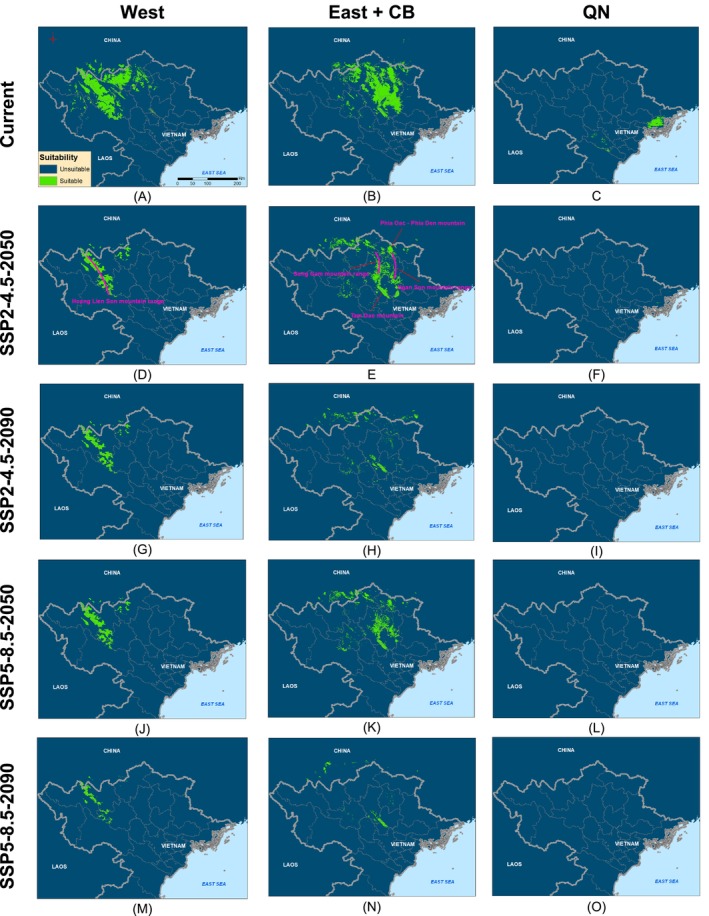
The suitable distribution of West, East + CB, and QN groups of Asian warty newts in northern Vietnam under climate change scenarios SSP2‐4.5 and SSP5‐8.5 in 2050 and 2090.

High values for KAPPA, the AUC, and TSS metrics were found in all models of the three groups (all values > 0.8; Figure S4), indicating strong predictive capacity for the selected algorithms. Under present climate conditions, the regions encompassing Yen Bai, Lao Cai, and Ha Giang in the western part of northern Vietnam were identified as particularly suitable for West. Conversely, the eastern region composed of Vinh Phuc, Thai Nguyen, Tuyen Quang, Bac Kan, and CB were particularly suitable for East + CB (Figure 7A,B). The areas predicted to be suitable for West and East + CB were similar in size, covering 12,456.18 km^2^ and 12,988.35 km^2^, respectively (Table 1). Conversely, the area predicted to be suitable for QN was mainly within QN Province and only approximately 1278.18 km^2^ in size (Figure 7C, Table 1).

**TABLE 1:** The changes in suitable area for main groups of Asian warty newts in northern Vietnam climate change scenarios SSP2‐4.5 and SSP5‐8.5 in 2050 and 2090 (unit: km^2^).

	Current (km^2^)	SSP2‐4.5‐2050			SSP2‐4.5‐2090			SSP5‐8.5‐2050			SSP5‐8.5‐2090		
		Stable (%)	Gain (%)	Loss (%)	Stable	Gain (%)	Loss (%)	Stable (%)	Gain (%)	Loss (%)	Stable (%)	Gain (%)	Loss (%)
West	12,456.18	33.45	0.10	66.55	28.60	0.08	71.40	29.61	0.07	70.39	12.28	0.00	87.72
East + CB	12,988.35	39.33	14.37	60.67	6.80	11.94	93.20	32.91	10.07	67.09	3.29	3.75	96.71
QN	1278.18	0.38	1.077	99.62	0.00	0.127	100	0.00	0	100	0.00	0	100

**FIGURE 8:**
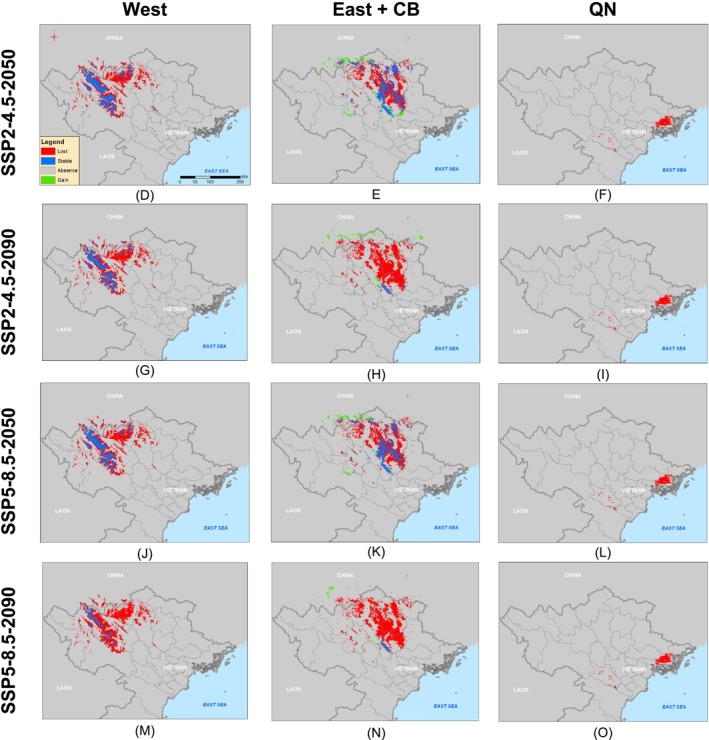
Estimation of suitable distribution change, in comparation to current suitable habitat (gain/loss), of West, East + CB, and QN groups of Asian warty newts in northern Vietnam under climate change scenarios SSP2‐4.5 and SSP5‐8.5 in 2050 and 2090.

We observed projected declines in the suitable distributions of all groups in response to climate change, except for East + CB under scenario SSP2‐4.5‐2050 (Table 1). In all scenarios, the expected decrease in the distribution of the West group was more pronounced than that of East (Figures 7, 8; Table 1). Specifically, the suitable habitat of West was projected to decrease by 66.55%–71.40% under scenario SSP2‐4.5 and by 70.39%–87.72% under scenario SSP5‐8.5. It should be noted that the suitable habitat gains of West is very limited by < 0.1%. Similarly, the suitable habitat for East + CB may decrease by 60.67% and 93.20% under the scenario SSP2‐4.5 in 2050 and 2090, respectively. Under scenario SSP5‐8.5, the suitable habitat for East + CB is likely to decrease by 67.09%–96.71%. However, the suitable habitat gains of East + CB were predicted at around 3.75%–14.37%. The impact of climate change on the suitable habitat for QN is also expected to be severe across all scenarios. In particular, QN could almost disappear under climate change scenario SSP5‐8.5‐2050 and SSP4‐8.5‐2090, with nearly 100% of suitable habitat lost (Figure 8; Table 1).

We also found shifts in the suitable distribution of all three primary groups of Asian warty newts toward higher altitudes under our climate change scenarios (Figure S5). The distributions in mountainous regions (e.g., the Hoang Lien Son and Tam Dao mountain ranges) were predicted to remain unaltered, suggesting that these regions may safeguard species from the effects of climate change (Figure 7). Furthermore, the centroids of suitable habitats for West and East + CB tended to shift northwest, whereas the centroid of suitable habitats for QN tended to shift northeast (Figure S6).

## 4 Discussion

PCA and ADMIXTURE analyses of nuDNA revealed three primary genetic clusters (West, East + CB, QN) of Asian warty newts in northern Vietnam. The discordance between the mtDNA and nuDNA SNP data for CB suggests contributions from distinct ancestral groups in East and QN. A close relationship between CB and East exists based on nuDNA SNP data. However, in mtDNA analyses, CB clustered with QN and GX in China, reflecting genetic introgression from *P. guangxiensis* (Tran, Vu, et al. 2023). Discordance in species delineations determined by mtDNA and nuDNA is common in salamanders and newts (Bisconti et al. 2018; Denton et al. 2014; Fisher‐Reid and Wiens 2011; Tomimori et al. 2023; Zieliński et al. 2013). In CB, mtDNA from the original newts may have been replaced entirely with mtDNA from *P. guangxiensis* by introgression.

In various urodeles, partial or complete replacement of mtDNA has been observed (Petit and Excoffier 2009; Toews and Brelsford 2012; Zieliński et al. 2013), and we here provide an example from Asian warty newts in northern Vietnam. Both a paleodistribution analysis of the last interglacial period and the last glacial maximum suggest that the mountainous regions of northern Vietnam likely served as refugia for these newts, potentially fostering introgression by gathering several lineages together (Tran, Vu, et al. 2023). Some of these lineages have probably interbred during glacial periods, and populations have expanded. They have become separated by geographical barriers, such as lowland areas, but have retained genetic purity in their nuDNA.

Interestingly, the morphology of CB was more similar to *P. deloustali* (= East) than that of *P. guangxiensis* (= QN; Tran et al. 2024). By modeling the suitable distributions of East and CB separately, Tran et al. (2024) also observed substantial ecological overlap. There are noteworthy similarities in the ecological niches of these two groups compared to the null distribution and thus, CB should be treated as *P. deloustali* .

Our study shows lower *F*_*ST*_ values between East and CB, which may be due to their geographical proximity based on the Mantel test (genetic differences are significantly correlated with geographic distance), reflecting the predominant influence of IBD on the population structure of these newts and being consistent with the small relative contribution of IBE found in the simulations performed. There is a large genetic difference between QN (including DS and KT) and other populations considered, supporting the hypothesis that QN forms a distinct taxon. This is also consistent with mtDNA data (Tran, Vu, et al. 2023). This considerable genetic difference is likely due to isolation resulting in restricted gene flow (Lovrenčić et al. 2022) and is supported by the divMigrate analyses, which revealed significant gene flow only from East to CB.

For *P. deloustali* , the introgressed CB group displayed higher genetic diversity than East or West. This higher genetic diversity is likely attributed to genomes being admixed with *P. guangxiensis*, similar to a newt population of *Lissotriton vulgaris* in northern Hungary (Herczeg et al. 2023). In the QN group ( *P. guangxiensis* ) there was greater genetic variation compared to West and East ( *P. deloustali* ). The low genetic diversity in West is due to geographic isolation (Tran, Vu, et al. 2023) and is revealed by divMigrate analyses (a measure of the absence of gene flow). Although the *F*_*IS*_ values of all populations in our study were low (< 0.15), there is a risk of inbreeding in the near future if the restricted gene flow persists (Keller and Waller 2002; Kenney et al. 2014). Both limited dispersal capacity and the fragmentation of suitable habitats (Matsui et al. 2006; Wells 2007; Figure 7) contributes to the isolation of these newt populations. However, we acknowledge that the number of variant sites in this study is limited due to the use of the MIG‐seq method. Here, we recommend that future studies incorporate a larger number of variant sites or integrate additional genomic data using methods such as RAD‐seq (Baird et al. 2008) or GRAS‐Di (Hosoya et al. 2019) to further validate and refine these relationships.

We evaluated the effects of climate change on the suitable habitats and genetic structure of populations of newts, *P. deloustali* and *P. guangxiensis* , in northern Vietnam. Overall, our findings suggest that climate change could decrease the geographical distribution of these newts, changes in the distribution of genetic clusters, and reductions in the size of areas available to these newts across all scenarios. Therefore, with rising temperatures and more severe weather patterns due to climate change, this will not be favorable for Asian warty newts. These results align with findings for other mountain salamanders and newts, and reductions in suitable habitats are an inevitable consequence of climate change for these species due to their sensitivity to environmental conditions and limited capacity for dispersal (Achour and Kalboussi 2020; Ashrafzadeh et al. 2019; Ebrahimi et al. 2022; Niknaddaf et al. 2023; Vaissi 2021).

Two examples of projected declines in suitable habitat for newts suggest a decline of about 20%–63% and 56%–98% in *Neurergus crocatus* and *N. kaiseri* , respectively (Ebrahimi et al. 2022; Ashrafzadeh et al. 2019). For much larger salamanders, such as *Andrias davidianus,* , suitable habitat could decrease by nearly 70% (Zhang et al. 2020). Furthermore, historical climate change has likely affected the diversification of the genus *Paramesotriton* (Luo et al. 2022). Therefore, we assume that other Asian warty newts in China will also be significantly affected by climate change. Additionally, the Asian warty newt is a stream‐dwelling species, meaning its suitable habitat is closely linked to stream systems. We recommend incorporating stream systems into the ENM analysis to enhance the accuracy of predicted suitable habitats and better assess the impact of climate change on its future distribution.

Our data show that the suitable habitat of the West group of *P. deloustali* is likely to be more severely affected by climate change than that of East + CB. Analyses of the spatial distribution of genetic clusters conditioned by environmental variation in these two groups suggested opposite trends, with the genetic clusters of East + CB expected to expand, but to decrease or remain stable in West. Surprisingly, all populations in East + CB displayed higher genetic diversity (*Ho*, *He*, and *Π*) compared to populations in West. East + CB was introgressed by genomic material originating from *P. guangxiensis* . In other studies, newt populations characterized by low genetic diversity may exhibit increased sensitivity to climate change (Abreu‐Jardim et al. 2021; Frankham 2005; Rizvanovic et al. 2019), but with introgression, there can be enhanced tolerance to environmental change, potentially benefiting populations by mitigating the effects of global warming (Brauer et al. 2023; De‐Kayne et al. 2022; Meier et al. 2019). Thus, the genetic admixture exhibited by East + CB may make this group less vulnerable to climate change. However, climate change‐adapted loci need to be identified to improve our understanding of climate adaptations and genomic vulnerability among populations.

Various species are expected to migrate to higher elevations due to global warming (Li et al. 2013; Wilson et al. 2005). Amphibians may relocate to cooler habitats within their existing range or migrate to higher elevations, and there are at least nine lowland amphibian species that have migrated upward by ≥ 500 m in Southeast Asia over the past 70 years (Bickford et al. 2010). Asian warty newts may also move to higher elevations, as seen in other urodeles that favor the cooler climates in montane habitats (Sparreboom 2014). In this study, we found that all three primary groups of Asian warty newts in northern Vietnam are predicted to relocate to higher elevations under all climate change scenarios, and these locations are similar to the refugia populated by newts during the glacial period (Tran, Vu, et al. 2023). The West group is expected to relocate to high elevations in the Hoang Lien Son and Tay Con Linh Mountains. The East + CB group may move to higher elevations in the mountain ranges of Song Gam and Ngan Son, Tam Dao, and the Phia Oac–Phia Den National Park, while the QN group is projected to relocate to the Tay Yen Tu Mountains.

Centroid migration analysis provides insight into changes in the suitable distributions of species. The centroid of the suitable range of Asian warty newts shifted due to the predicted impact of climate change (Wang et al. 2024). The suitable habitat of the West and East + CB groups shifted northwestward, whereas the QN group shifted southwestward. The tendencies of newt relocation suggest that their original habitats may no longer be suitable, and therefore, biodiversity corridors are critical to enable species to migrate toward suitable habitats and enhance gene flow among populations responding to climate change (Sahlean et al. 2020; Vu et al. 2020). These corridors are particularly crucial for species with limited dispersal capacity, such as many amphibians (Sahlean et al. 2020). In northern Vietnam, there are three biodiversity corridors that could link protected areas and mitigate the impact of climate change on biodiversity. These are the Northeast Corridor (Ba Be National Park [NP] to Du Gia–Cao Nguyen Da Dong Van NP), the Northwest Corridor (Cuc Phuong NP to Ngoc Son–Ngo Luong Nature Reserve [NR]), and the Northern Coastal Corridor (Dong et al. 2013). After considering the suitability of habitats projected by ENM and gene flow within the East + CB group, we propose a new corridor from Than Sa—Phuong Hoang NR through the Ngan Son Range (Figure 7) to the Phia Oac Mountains (see schematic diagram of the corridor in Figure S7). This corridor could also connect Kim Hy NR and Phia Oac–Phia Den NP. Once this corridor is established, it may play an important role in safeguarding threatened species, including Asian warty newts, from the threats posed by climate change.

### 4.1 Conservation Implications

Our study revealed the introgression between *P. deloustali* and *P. guangxiensis* in the CB population (Cao Bang Province, northern Vietnam), which exhibited higher genetic diversity. This indicated that hybridization events may enhance genetic diversity and adaptation to local environments by newts. More intensive monitoring programs should be implemented to track genetic diversity and the hybrid zones of this population. Additionally, conservation strategies should prioritize populations with greater genetic diversity and focus on mitigating threats to genetic integrity.

The projected shifts to suitable habitat to higher elevations because of climate change highlights the importance of conservation planning. Our results suggested that the high mountain areas of northern Vietnam possibly serve as refugia for warty newts in northern Vietnam. These areas facilitated genetic exchange and lineage diversification (Tran, Vu, et al. 2023) and these mountainous habitats may once again serve as critical refugia. Therefore, conservation efforts should focus on protecting these highland habitats, especially focusing on streams, and ensuring connections among refugia through biodiversity corridors. These conservation plans should be accompanied by policies to mitigate anthropogenic impacts, such as deforestation and agricultural expansion, and pollution. The severe reduction and potential disappearance of suitable habitat for the QN group under all future climate scenarios also mean there is an urgent need for conservation efforts. Priorities should include habitat preservation, ecosystem restoration, the establishment of climate‐resilient refuges, and ex‐situ measures like breeding programs to safeguard the population.

In conclusion, our population genetics analysis identified three primary groups of Asian warty newts in northern Vietnam: West, East + CB, and QN. Contrary to previous assumptions that the CB group belonged to the *P. guangxiensis* population (West), our SNP data suggest that it should be classified as a population of *P. deloustali* with introgressive mtDNA from *P. guangxiensis* . There is restricted gene flow among populations of Asian warty newts, and both spatial genetic clusters and the suitable habitats for them are vulnerable to climate change impacts. Establishing biodiversity corridors connecting the high mountains of northern Vietnam is a critical measure to safeguard the Asian warty newts from extinction.

## Author Contributions

**Dung Van Tran:** conceptualization (lead), formal analysis (lead), funding acquisition (lead), investigation (lead), methodology (lead), validation (lead), visualization (lead), writing – original draft (lead), writing – review and editing (lead). **Tomoya Suzuki:** methodology (equal), visualization (equal), writing – review and editing (equal). **Ibuki Fukuyama:** methodology (equal), visualization (equal), writing – review and editing (equal). **Ricardo J. Vera:** methodology (equal), writing – review and editing (equal). **Kanto Nishikawa:** conceptualization (equal), investigation (equal), project administration (equal), supervision (lead), writing – review and editing (equal).

## Conflicts of Interest

The authors declare no conflicts of interest.

## Supporting information


Data S1.

## Data Availability

The occurrence locations, environmental data, and code used for Species Distribution Models were provided in the link: https://figshare.com/s/8cc7299463ef121a6d5b. The data and code used for running n‐dimensional hypervolumes were provided in the link: https://figshare.com/s/c6c5ad0b8eb8ed2c9a34. We deposited the raw sequence reads of MIG‐seq data in the DNA Data Bank of Japan (DDBJ) Sequence Read Archive (DRA) under accession number DRA019371 (BioProject ID: PRJDB18910; BioSample ID: SAMD00823858‐SAMD00823919).
