# Supplementary material for: Population Genetics Provides Insights Into the Impact of Future Climate Change on the Genetic Structure and Distribution of Asian Warty Newts (Genus Paramesotriton)
Source: Ecol Evol. 2025 Apr 3;15(4):e71054. doi: 10.1002/ece3.71054 (PMC11968420; doi:10.1002/ece3.71054)
Supplement: Supplementary file 1 — Data S1. [file ECE3-15-e71054-s001.docx]

**SUPPORTING INFORMATION**

**Table S1. The number of samples of each population using for MIG-Seq**

| **No** | **Pop** | **Location** | **Number of samples using for MIG-seq** |
| --- | --- | --- | --- |
| 1 | NK | Nam Khanh commune, Bac Ha district, Lao Cai province, Vietnam | 4 |
| 2 | NC | Na Chi commune, Xin Man district, Ha Giang province, Vietnam | 6 |
| 3 | DQ | Duong Quy commune, Van Ban district, Lao Cai province, Vietnam | 3 |
| 4 | NCo | Nam Co commune, Mu Cang Chai district, Yen Bai province, Vietnam | 6 |
| 5 | XT | Xuan Tam commune, Van Yen district, Yen Bai province, Vietnam | 5 |
| 6 | QT | Quang Thanh commune, Nguyen Binh district, Cao Bang province, Vietnam | 9 |
| 7 | DP | Don Phong commune, Bach Thong district, Bac Kan province, Vietnam | 9 |
| 8 | DD | Dai Dinh district, Tam Dao district, Vinh Phuc province, Vietnam | 9 |
| 9 | HH | Hop Hoa commune, Son Duong district, Tuyen Quang province, Vietnam | 3 |
| 10 | DS | Dong Son commune, Ha Long district, Quang Ninh province, Vietnam | 6 |
| 11 | KT | Ky Thuong commune, Ha Long district, Quang Ninh province, Vietnam | 2 |
| **Total** | | | **62** |

**Table S2. The selected environmental variables and VIF values**

| **No** | **Variables** | **VIF** |
| --- | --- | --- |
| 1 | Bio1 | 1.600905 |
| 2 | Bio2 | 2.286694 |
| 3 | Bio7 | 2.816285 |
| 4 | Bio12 | 2.518097 |
| 5 | Bio15 | 4.038963 |
| 6 | Forest | 1.743153 |
| 7 | Human footprint | 1.663004 |

**Table S3. Estimated cross-validation (CV) error of ADMIXTURE analysis for K = 2-11**

| CV error (K=2): 0.38748 |
| --- |
| **CV error (K=3): 0.34938** |
| CV error (K=4): 0.37471 |
| CV error (K=5): 0.36091 |
| CV error (K=6): 0.35725 |
| CV error (K=7): 0.41977 |
| CV error (K=8): 0.41219 |
| CV error (K=9): 0.45434 |
| CV error (K=10): 0.46888 |
| CV error (K=11): 0.49933 |

**Table S4. The pairwise *F_ST_* (below), and geographic distance (above) among the populations of the Asian warty newts in northern Vietnam (The distance unit: km)**

| **Group** |  | **East** | | | **West** | | | | | **QN** | | **CB** |
| --- | --- | --- | --- | --- | --- | --- | --- | --- | --- | --- | --- | --- |
|  | ***Pops*** | ***DD*** | ***HH*** | ***DP*** | ***NC*** | ***Nco*** | ***NK*** | ***XT*** | ***DQ*** | ***DS*** | ***KT*** | ***QT*** |
| **East** | ***DD*** | 0 | 23.4 | 88.6 | 164.4 | 140.5 | 168.7 | 122 | 163.2 | 143.4 | 163 | 127.6 |
|  | ***HH*** | 0.052 | 0 | 77.3 | 141 | 120.2 | 145.3 | 101.6 | 141.6 | 166.7 | 187.9 | 119.8 |
|  | ***DP*** | 0.110 | 0.141 | 0 | 139.2 | 159 | 147.9 | 140.1 | 170.2 | 169.6 | 187.3 | 40.3 |
| **West** | ***NC*** | 0.489 | 0.444 | 0.416 | 0 | 75.2 | 11.4 | 72.7 | 61.4 | 293.3 | 313.9 | 142.6 |
|  | ***Nco*** | 0.491 | 0.443 | 0.423 | 0.076 | 0 | 67.5 | 20.2 | 26.2 | 286.2 | 308.2 | 183.8 |
|  | ***NK*** | 0.438 | 0.369 | 0.367 | 0.054 | 0.051 | 0 | 69.0 | 52.7 | 307.8 | 327.1 | 164.6 |
|  | ***XT*** | 0.466 | 0.410 | 0.400 | 0.116 | 0.045 | 0.057 | 0 | 41.5 | 261.4 | 281.4 | 162.2 |
|  | ***DQ*** | 0.460 | 0.421 | 0.395 | 0.145 | 0.073 | 0.076 | 0.071 | 0 | 308.2 | 328.2 | 192.2 |
| **QN** | ***DS*** | 0.517 | 0.440 | 0.462 | 0.544 | 0.547 | 0.492 | 0.507 | 0.506 | 0 | 21.3 | 192.6 |
|  | ***KT*** | 0.555 | 0.513 | 0.481 | 0.606 | 0.606 | 0.563 | 0.589 | 0.590 | 0.206 | 0 | 204.7 |
| **CB** | ***QT*** | 0.221 | 0.147 | 0.167 | 0.383 | 0.379 | 0.305 | 0.333 | 0.333 | 0.353 | 0.335 | 0 |

**Table S5. The pairwise *F_ST_* (below) among four main groups of the Asian warty newts in northern Vietnam**

|  | **East** | **West** | **QN** | **CB** |
| --- | --- | --- | --- | --- |
| **East** | 0 |  |  |  |
| **West** | 0.40 | 0 |  |  |
| **QN** | 0.45 | 0.54 | 0 |  |
| **CB** | 0.19 | 0.43 | 0.33 | 0 |

**Table S6. Relative effects of IBD and IBE for each species**

| **Species** | **aD** | **aE elevation** | **aE annual temperature** | **aE annual precipitation** |
| --- | --- | --- | --- | --- |
| *P. delousteli* | 3.517 × 10^-3^ (3.445 × 10^-3^ - 3.589 × 10^-3^) | 4.754 × 10^-5^ (4.545 × 10^-5^ - 4.963 × 10^-5^) | 8.786 × 10^-3^ (8.608 × 10^-3^ - 8.963 × 10^-3^) | 1.970 × 10^-3^ (1.817 × 10-3 - 2.123 × 10-3) |
| *P. guangxiensis* | 6.739 × 10^-3^ (6.643 × 10^-3^ - 6.835 × 10^-3^) | 2.504 × 10^-3^ (2.473 × 10^-3^ - 2.536 × 10^-3^) | 0.651 (0.642 - 0.659) | 1.727 × 10^-4^ (1.699 × 10^-4^ - 1.755 × 10^-4^) |

**Table S7. The niche differentiation among realized niche of the West, East + CB, and QN group from n-dimensional hypervolumes**

| **Niche differentiation** | | |
| --- | --- | --- |
|  | West | East + CB |
| East + CB | 0.990 |  |
| QN | 1.000 | 0.999 |
|  |  |  |
| **Niche shifts** | | |
|  | West | East + CB |
| East + CB | 0.079 |  |
| QN | 0.016 | 0.269 |
|  |  |  |
| **Niche expansion/contraction (net differences)** | | |
|  | West | East + CB |
| East + CB | 0.911 |  |
| QN | 0.984 | 0.730 |
| **Intersection** | | |
|  | West | East + CB |
| East + CB | 3078422.494 |  |
| QN | 25805.613 | 4700.369 |
| **Sorensen similarity index** | | |
|  | West | East + CB |
| East + CB | 0.0256314556 |  |
| QN | 0.0002239456 | 0.0006993777 |


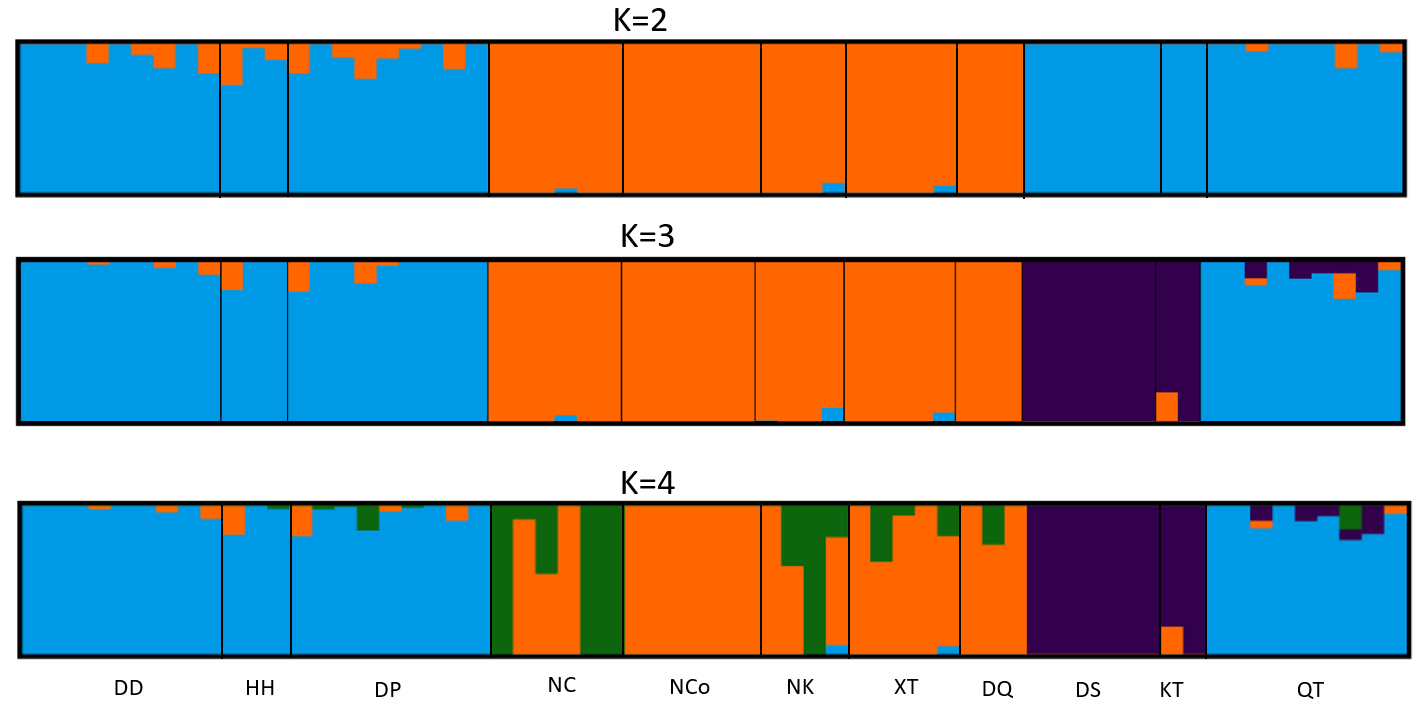


**Fig. S1. The ADMIXTURE analysis for K =2, 3, 4 from MIG-seq data of Asian warty newts in northern Vietnam**


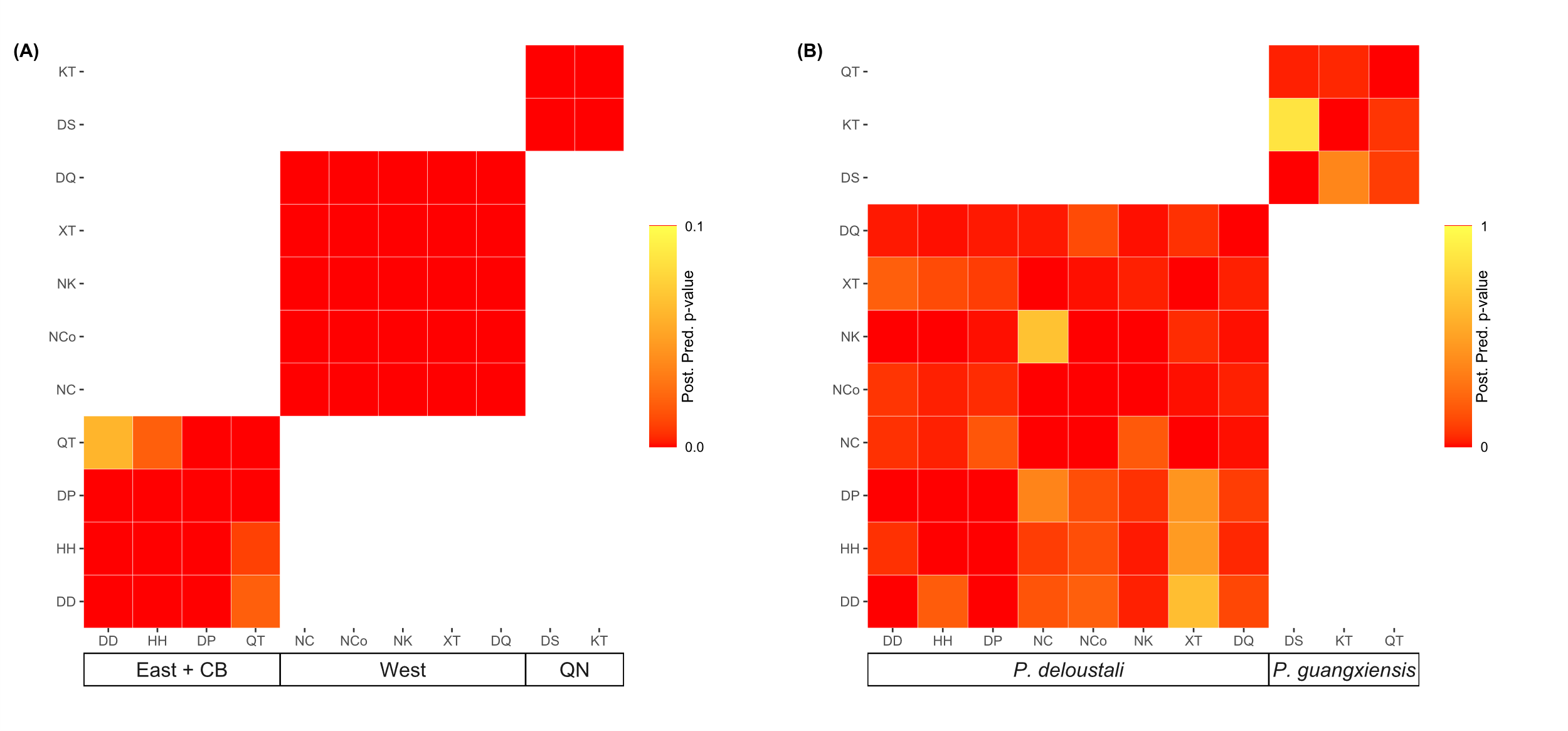


**Fig. S2. Performance of BEDASSLE models evaluated by posterior predictive p-value on a scale from 0 to 1 comparing (A) genetics clusters and (B) species. Higher p-values indicate better model fit. Upper and lower diagonal values correspond to models without and with overdispersion, respectively.**


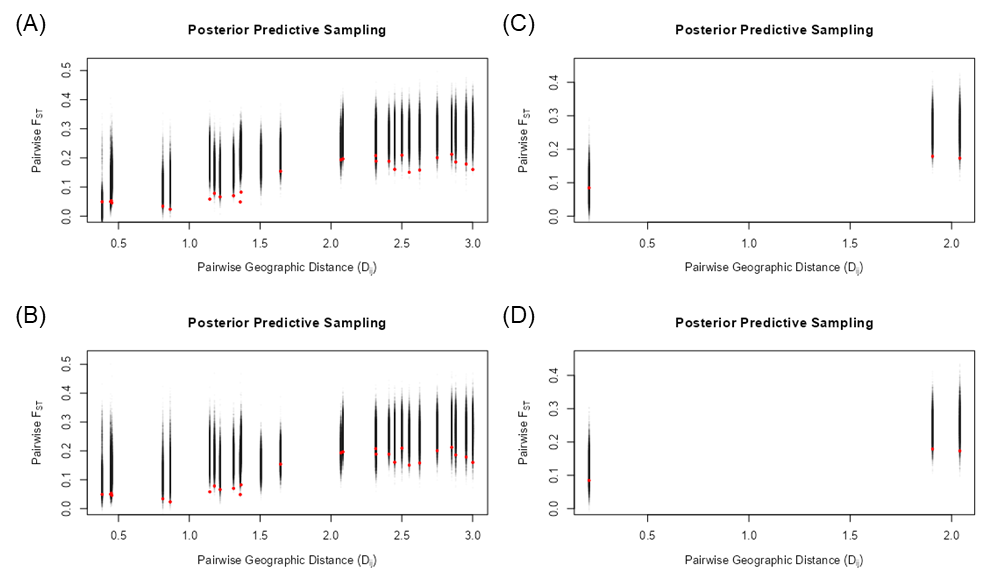


**Fig. S3. Posterior predictive sampling for within species analysis. Models for *P. deloustali* (A) without and (B) with beta-binomial extension and for *P. guangxiensis* (C) without and (D) with beta-binomial extension.**

| West group | 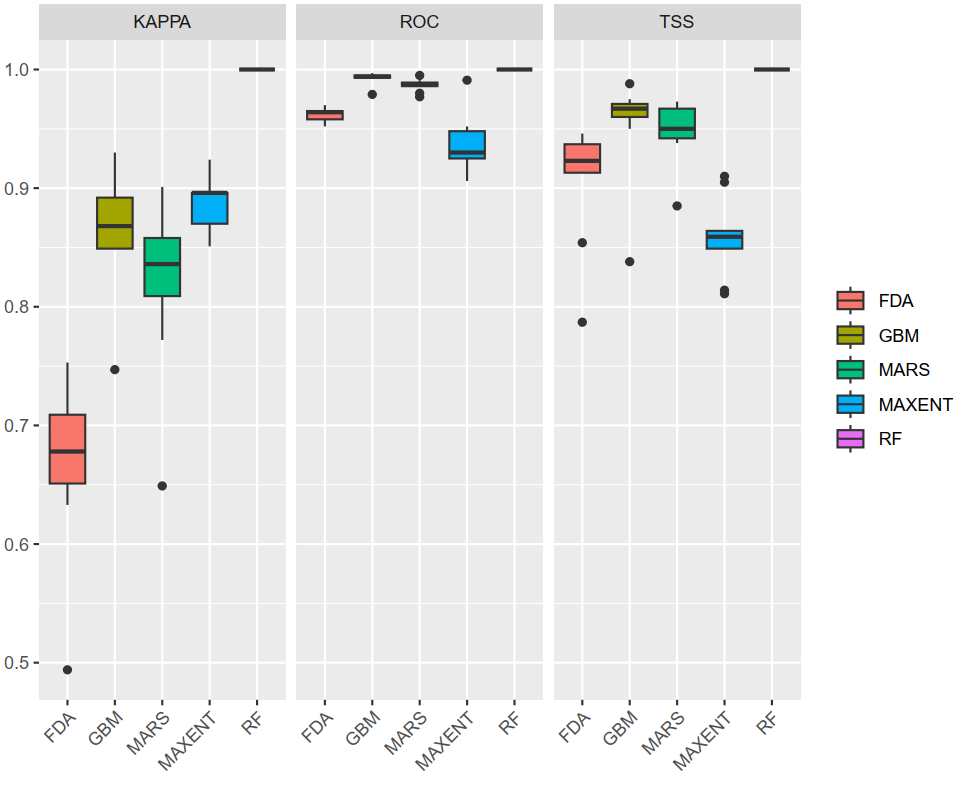 |
| --- | --- |
| East + CB group | 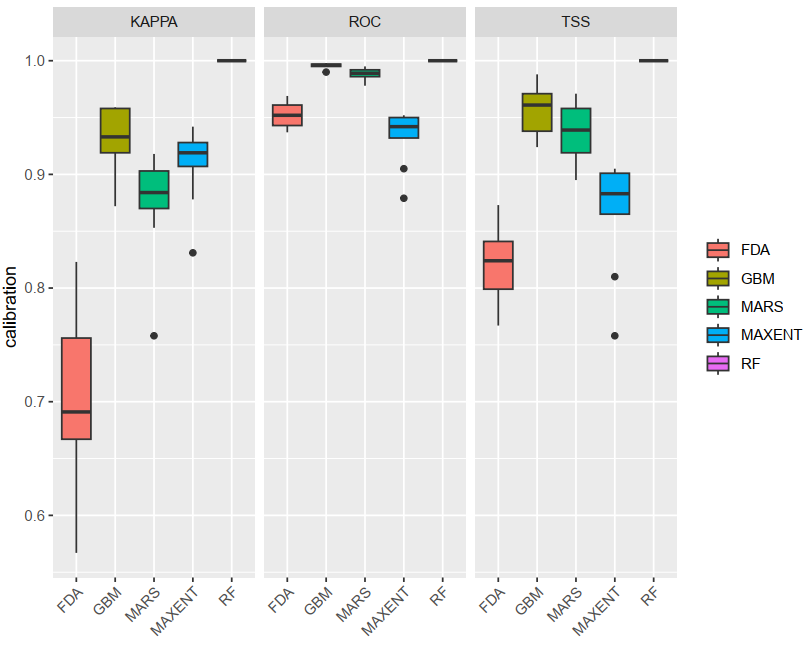 |
| QN | 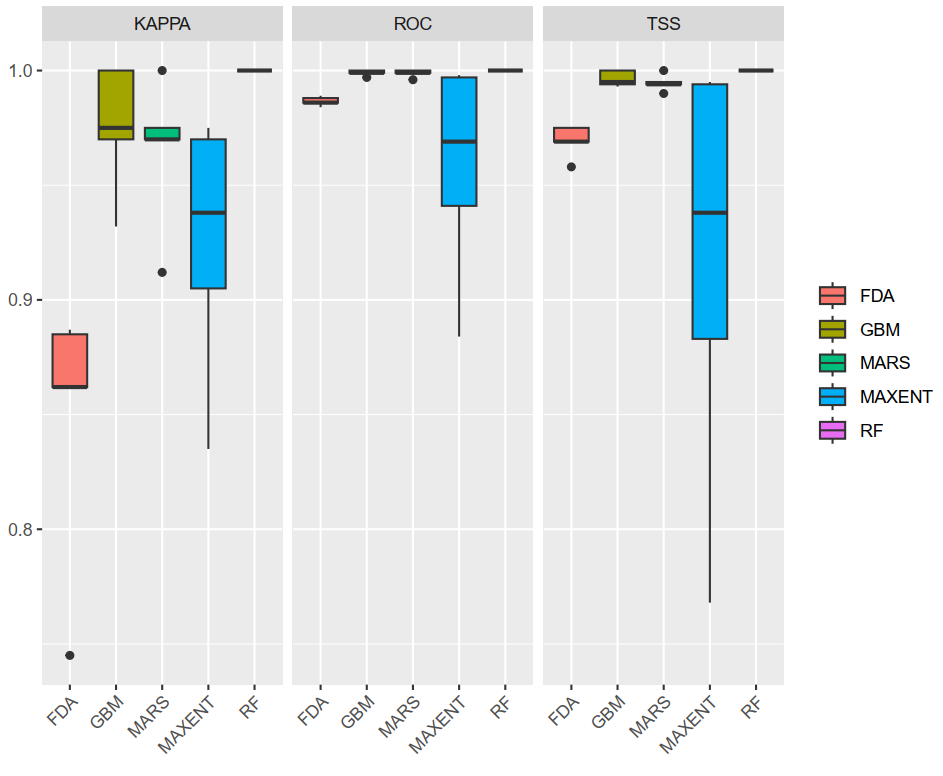 |
|  |  |

**Fig. S4. Cohen's Kappa (KAPPA), ROC curve (AUC), True skill statistic (TSS) of five used algorithms predicting the distribution of Asian warty newts in northern Vietnam**
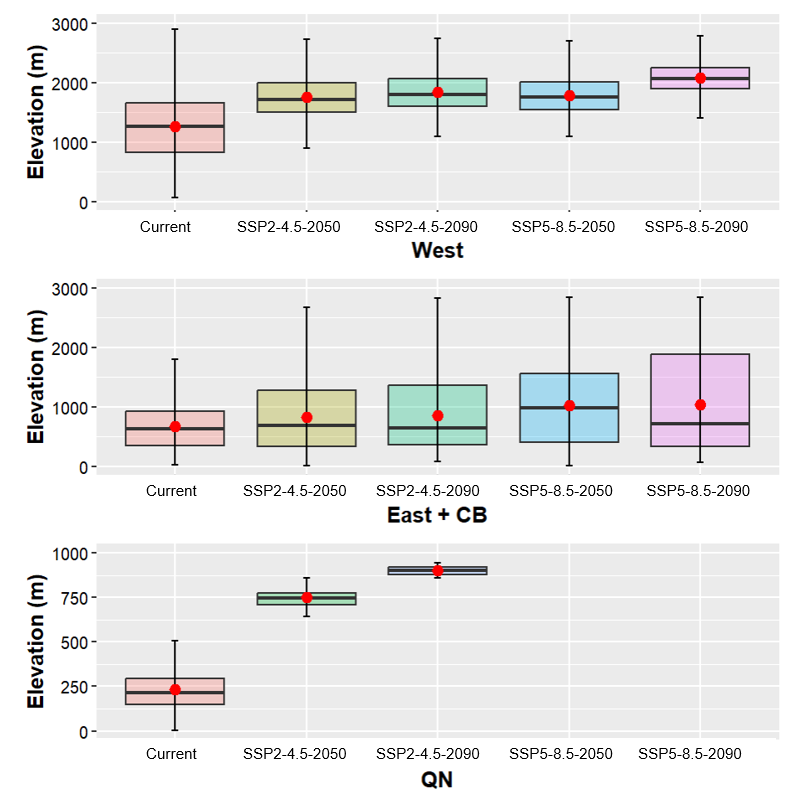


**Fig. S5. The elevational shift of four main group of Asian warty newts (West, East + CB, and QN) under climate change scenarios**


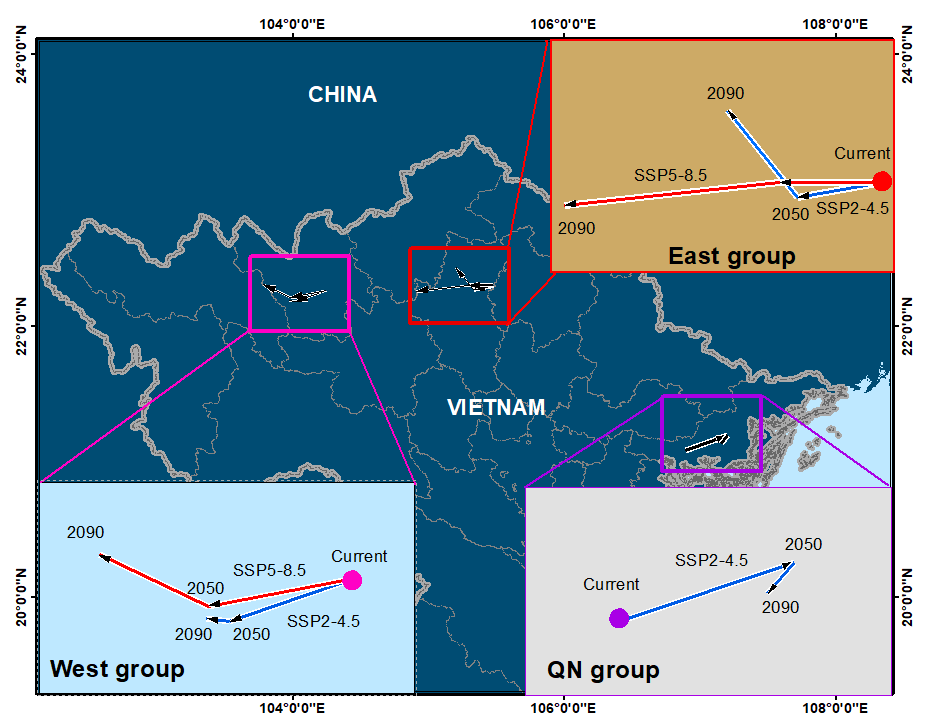


**Fig S6. The core distributional shifts of suitable habitat in Asian warty newts (West, East + CB, and QN) under climate change scenarios**


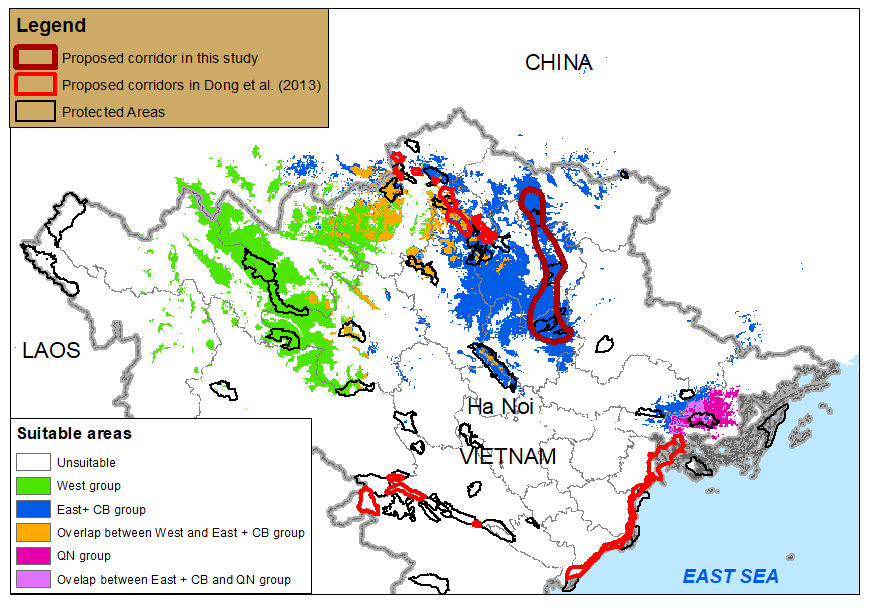


**Fig S7. The schematic diagram of the ecological corridors proposed by Dong et al. (2013) and by the study.**
